# Solvent-Free Oil-Based Extraction and Microencapsulation of Lutein from Marigold (*Calendula officinalis*)

**DOI:** 10.3390/molecules31101649

**Published:** 2026-05-13

**Authors:** Aleksander Wieland, Marcin A. Kurek

**Affiliations:** 1 Faculty of Human Nutrition, Warsaw University of Life Sciences (WULS-SGGW), Nowoursynowska 159 c Street, 02-776 Warsaw, Poland; s223075@sggw.edu.pl; 2 Department of Technique and Food Product Development, Institute of Human Nutrition Sciences, Warsaw University of Life Sciences (WULS-SGGW), Nowoursynowska 159 c Street, 02-776 Warsaw, Poland

**Keywords:** lutein, microencapsulation, SEM, starch, maltodextrin, oil extraction, solvent-free extraction

## Abstract

Lutein is a highly unstable antioxidant traditionally extracted using toxic solvents. To address this, this study evaluates the microencapsulation of marigold (*Calendula officinalis*) lutein utilizing a solvent-free, oil-based extraction approach as a potentially greener alternative to conventional organic solvent extraction. Lutein was extracted via Soxhlet, ultrasound-assisted extraction, and oil extraction using sunflower, corn, and grape seed oils. Emulsions were formulated with maltodextrin combined with gum arabic, tapioca starch, or waxy maize starch, and spray-dried. The resulting microcapsules demonstrated favorable moisture contents (0.98% to 3.43%) and high solubility (70.5% to 85.81%). Encapsulation efficiency ranged from 34.98% to 56.59%, peaking in formulations utilizing waxy maize starch and sunflower oil. Flowability was restrictive across all powders, indicated by elevated Carr’s Compressibility Index values. Scanning electron microscopy revealed predominantly smooth, spherical particles measuring less than 6 µm. Notably, while unencapsulated sunflower oil extract exhibited the highest lutein concentration, microcapsules containing grape seed oil showed relatively higher lutein concentration in the final product. This observation may be related to differences in oil composition; however, further studies are required to confirm the underlying mechanisms.

## 1. Introduction

Lutein is a natural yellow pigment belonging to the xanthophyll group of carotenoids, which can be found in various plants, fruits, and vegetables [1,2]. It plays a crucial role in human health, mostly because it accumulates in the macula of the human eye and acts as an antioxidant [3,4]. Lutein helps filter harmful blue light and protects retinal cells from oxidative stress, significantly reducing the risk of age-related macular degeneration and cataracts [3,5]. Because the human body cannot produce lutein on its own, it must be delivered through our daily diet or dietary supplements [6]. However, a standard diet usually does not provide enough of this compound to achieve optimal health benefits [4]. The recommended minimum daily intake associated with health benefits, such as a reduced risk of age-related macular degeneration, is 6 mg of lutein [6,7,8]. Currently, commercial production of lutein primarily relies on extraction from marigold flowers [6]. Traditionally, this process uses toxic organic solvents, such as hexane, which creates serious environmental and health concerns [9]. Conventional solvent extraction poses several risks, including potential toxic residues, high energy consumption, occupational hazards for factory workers, and the generation of problematic organic waste [10,11]. Researchers and the industry must explore non-toxic alternative solvents, supercritical fluid extraction, and ultrasound-assisted techniques to make the extraction process more environmentally friendly, scalable, and safer for consumers [12,13].

Even when successfully and sustainably extracted, lutein faces another major challenge because it is highly unstable. Its chemical structure contains many double bonds, making it extremely sensitive to heat, light, oxygen, and acidic environments [14,15]. This instability causes lutein to easily degrade during food processing and long-term storage [15]. Additionally, lutein is a fat-soluble compound with very poor water solubility, which limits its absorption in the human gastrointestinal tract and results in low bioavailability [8]. These factors make the direct application of free lutein in dietary supplements very difficult and inefficient.

To overcome these obstacles and create effective supplements, microencapsulation is widely used as a reliable protection strategy [15]. This technology involves enclosing the sensitive lutein core within a protective wall material that separates the pigment from harmful environmental factors and extends its shelf life [14]. Spray drying is the most common technique for microencapsulation of lutein [4]. Choosing the right wall material is critical for the success of this process. Maltodextrin is often chosen for this purpose due to its low cost, high water solubility, low viscosity, and ability to protect encapsulated compounds from oxidation [1]. A serious drawback of maltodextrin itself, however, is its poor emulsifying properties, which make it rarely used on its own, and it is usually combined with other biopolymers, like starch, to achieve optimal coating effects [16,17].

To overcome both the environmental hazards of traditional extraction and the inherent instability of the pigment, sustainable, non-toxic processing must be paired with reliable and established microencapsulation techniques. In this context, lutein and other carotenoids can be extracted directly into edible vegetable oils and incorporated into spray-dried microcapsules, which improves their handling and helps to limit thermal and light-induced degradation. Most existing research, however, either optimizes general extraction conditions or focuses on standard spray-drying parameters, lacking a comprehensive comparison of different edible oils used as continuous carriers throughout this integrated process.

To address this technological gap, sunflower, corn, and grape seed oils were deliberately selected as extraction media and lipid cores in this study. These specific oils were chosen due to their commercial relevance and distinct compositional profiles, which may influence their performance as non-toxic solvents. However, it remains underexplored how these distinct lipid matrices compare against each other regarding extraction efficiency and subsequent protection within the powder formulation. Therefore, this study tests the hypothesis that the specific physicochemical and phytochemical profile of the chosen carrier oil can significantly differentiate not only the extraction yield of lutein from the botanical matrix but also its relative preservation within the spray-dried microcapsules. Addressing this, the present study utilizes these selected oil matrices alongside maltodextrin combined with waxy maize or tapioca starches, ultimately aiming to provide an antioxidant formulation without the use of hazardous chemical solvents.

## 2. Results

### 2.1. Physical Properties

#### 2.1.1. Moisture Content

The moisture content of the microcapsules synthesized during this research ranged from 0.98% to 3.43%. Statistical evaluation using the Tukey HSD test revealed significant differences among the formulations, categorizing the results into five distinct homogeneous groups. The control formulation (CON) exhibited the highest moisture retention at 3.43% ± 0.06%, placing it exclusively in group ‘a’. Conversely, the lowest moisture levels, reaching a minimum of 0.98% ± 0.02%, were observed in the STA, CTA, and CHS samples, which were collectively assigned to group ‘e’. Formulations characterized by intermediate moisture values included ETA (2.45% ± 0.05%, group ‘b’), a cluster comprising EHS, SHS, and GHS (all at 1.96% ± 0.04%, group ‘c’), and finally formulation GTA (1.47% ± 0.03%, group ‘d’). Interestingly, microcapsules containing extracts obtained via oil-based extraction exhibited moisture levels equal to or lower than those obtained with Soxhlet and ultrasound-assisted extraction (UAE) methods (Table 1).

#### 2.1.2. Solubility

The solubility of the microcapsules, quantified as a percentage of the initially added powder, ranged from a minimum of 70.5 ± 5.61% (CON) to a maximum of 85.81 ± 0.24% (CHS). The Tukey HSD test revealed no significant differences (*p* > 0.05) among the experimental formulations. The data indicate that despite using different wall materials and extraction methods, all experimental powders achieved solubility comparable to the control system (Table 1).

#### 2.1.3. Oil Content and Encapsulation Efficiency

The unencapsulated surface oil content of the microcapsules obtained in this study is between 13.98 and 20.57 g/100 g. The values obtained for the CON and ETA (20.48 ± 0.50 g/100 g and 20.57 ± 1.50 g/100 g, respectively) groups are the highest, although they differ significantly only from the SHS and CHS groups. The least amount of oil was found on the surface of the SHS group (13.98 ± 3.00 g/100 g), which correspondingly resulted in the highest encapsulation efficiency (56.59 ± 10.44%). Interestingly, samples with a maize starch matrix have lower surface oil values than their tapioca starch counterparts, although these differences are not always statistically significant (Table 2).

For total oil content, no statistically significant differences were found.

In terms of encapsulation efficiency, the SHS microcapsules achieved the highest value (56.59 ± 10.44%), while the ETA samples had the lowest (34.98 ± 5.80%). A statistically significant difference occurred only between these two groups (Table 2).

#### 2.1.4. Bulk Density, Tapped Density, Carr’s Compressibility Index, and Hausner Ratio

The highest bulk density, significantly different from that of other formulations, was achieved for the control sample (CON). This sample also obtained the highest tapped density, the lowest Carr’s Index, and the lowest Hausner ratio (Table 3).

Another distinguishing sample is CTA, which exhibited the poorest flowability parameters. It can also be noted that microcapsules obtained by ultrasound-assisted extraction (ETA and EHS) showed more favorable values for tapped density, Carr’s Index, and Hausner Ratio. However, the differences from the solvent-free extraction groups were statistically significant only in some cases (Table 3).

### 2.2. Scanning Electron Microscopy (SEM)

#### 2.2.1. Surface Morphology

**Figure 1:**
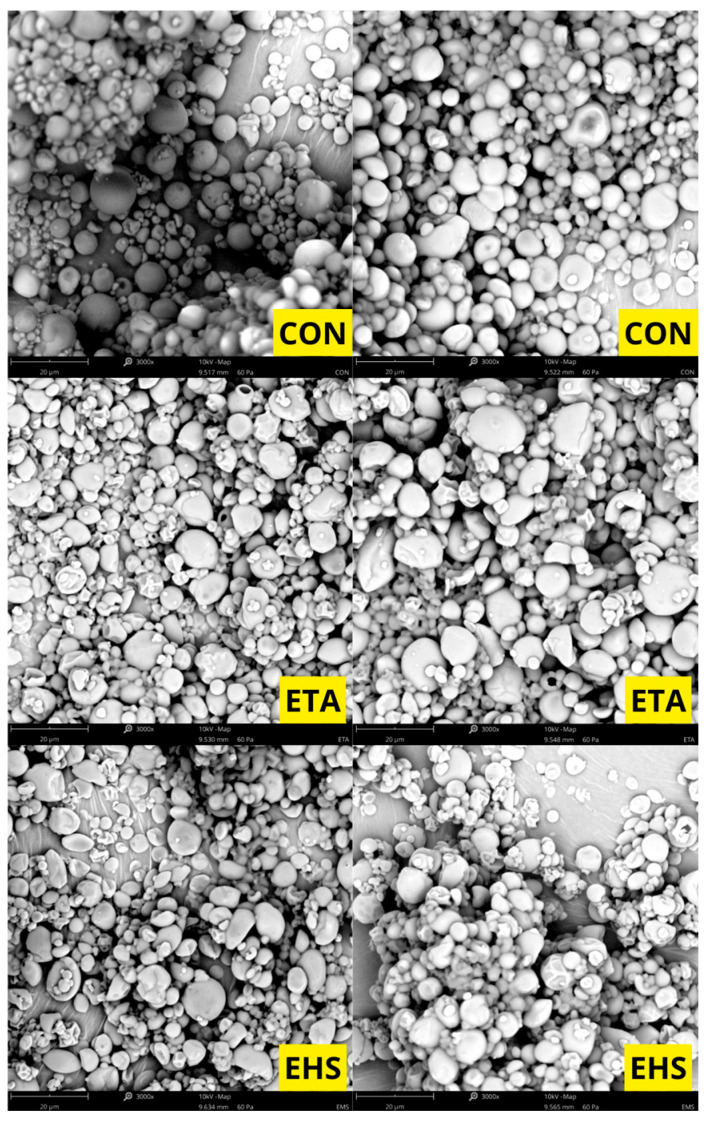
Scanning electron microscopy photographs of samples CON, ETA, and EHS.

**Figure 2:**
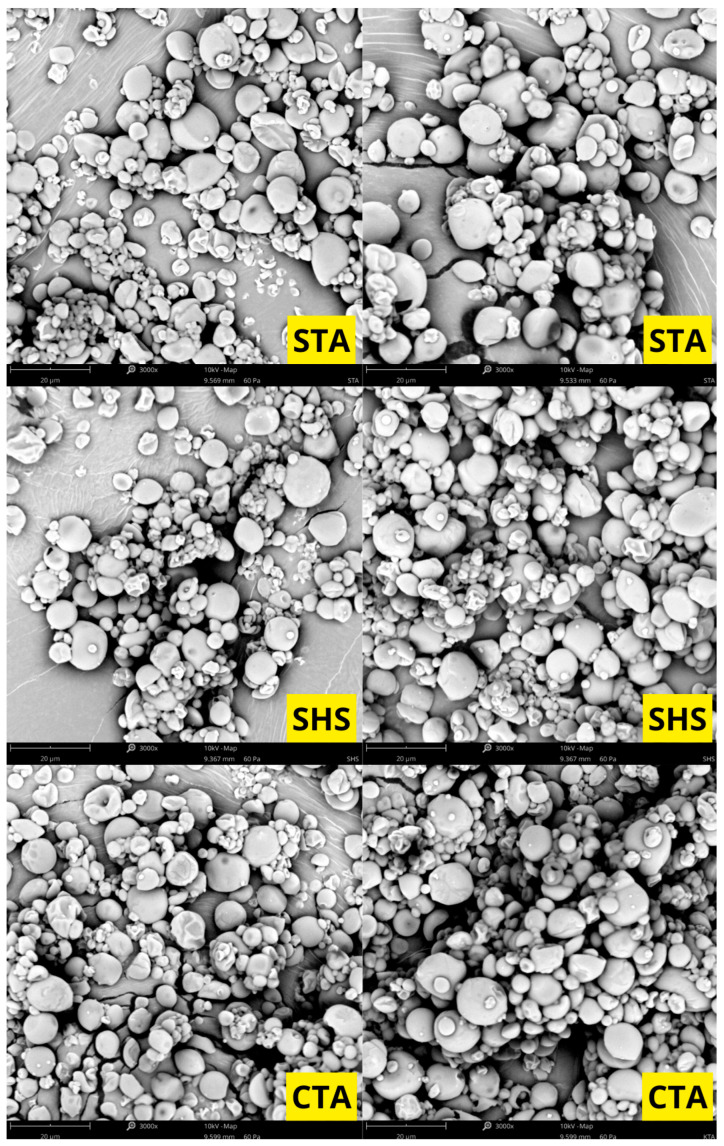
Scanning electron microscopy photographs of samples STA, SHS, and CTA.

**Figure 3:**
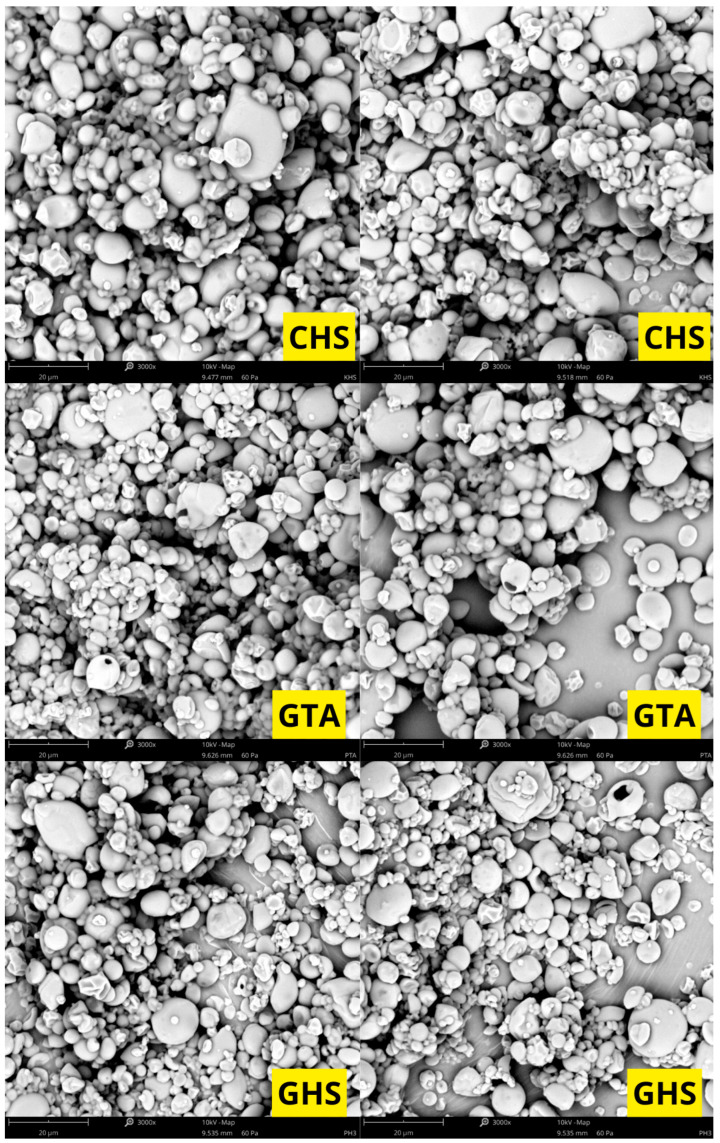
Scanning electron microscopy photographs of samples CHS, GTA, and GHS.

Based on the scanning electron microscopy (SEM) images, the morphology of the investigated samples was evaluated. The micrographs reveal that the material consists of particles (granules) with diverse shapes—spherical, oval, and polygonal structures with rounded edges are dominant. The surface of the vast majority of particles is relatively smooth, free of deep cracks or fissures. A strong polydispersity of the tested material is noticeable in the micrographs; alongside a numerous group of smaller particles, there are individual, distinctly larger granules, and in some areas, small fragments and minor agglomerates are visible. The particles are characterized by close packing on the specimen stub (Figure 1, Figure 2 and Figure 3).

#### 2.2.2. Image Segmentation and Particle Size Distribution

To perform a quantitative assessment of granule size, the raw SEM images were processed using instance segmentation algorithms. The colored masks confirm the effective detection and separation of individual particle boundaries, enabling reliable counting of objects and determination of their diameters (in µm).

**Figure 4:**
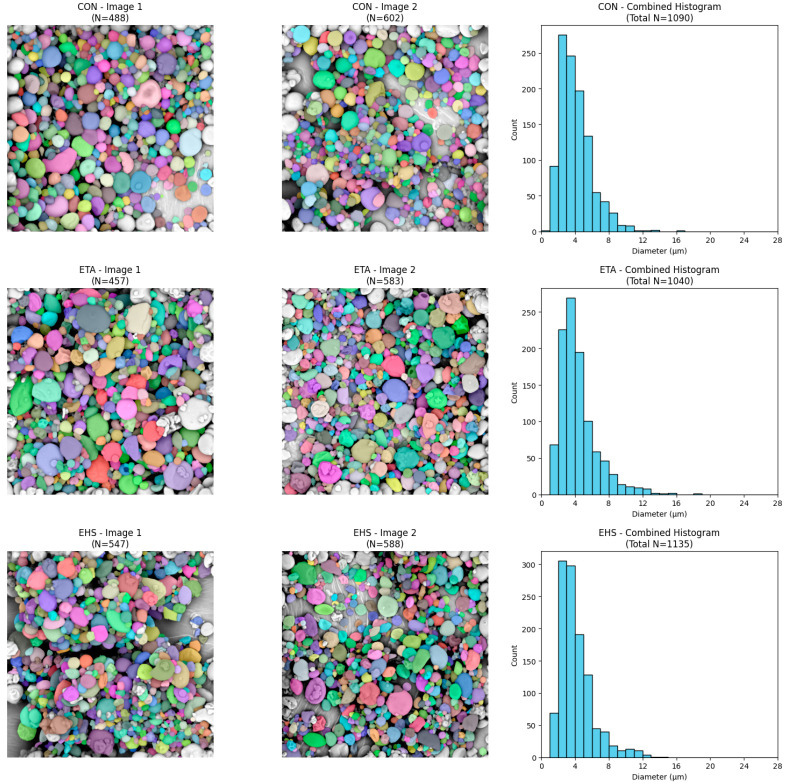
Representative microcapsule segmentation and corresponding size distribution for samples CON, ETA, and EHS.

**Figure 5:**
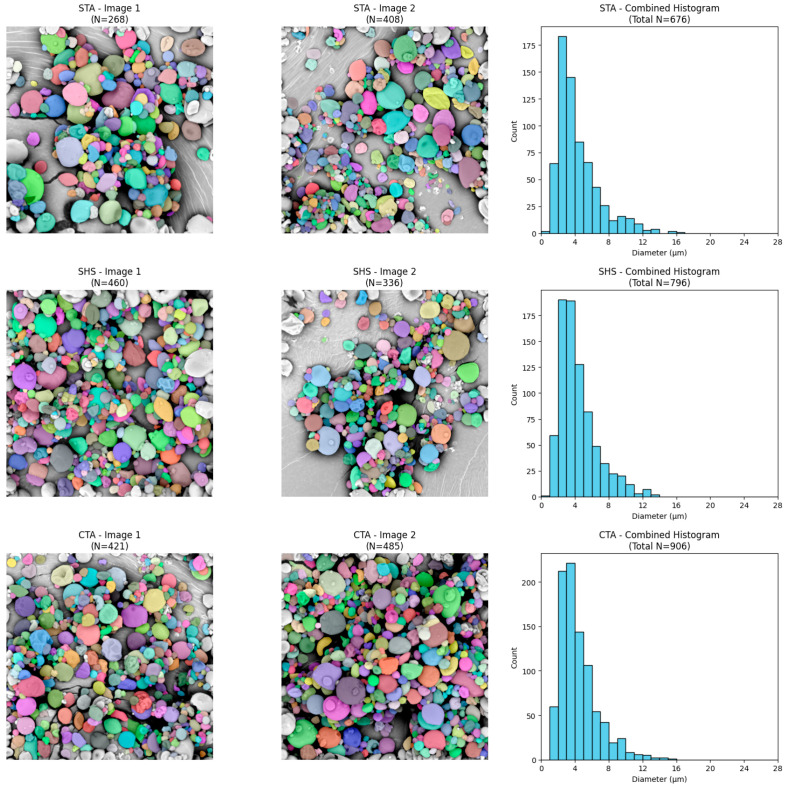
Representative microcapsule segmentation and corresponding size distribution for samples STA, SHS, and CTA.

**Figure 6:**
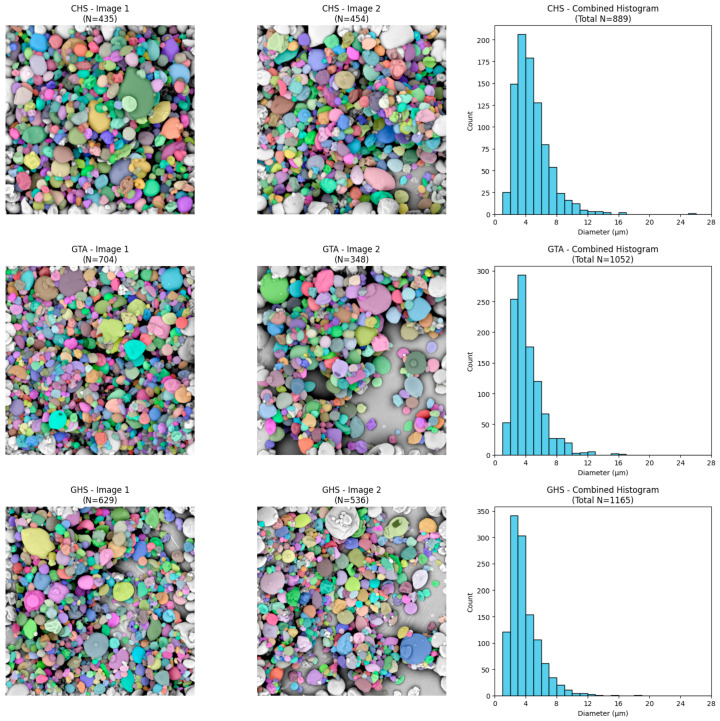
Representative microcapsule segmentation and corresponding size distribution for samples CHS, GTA, and GHS.

An analysis of the generated histograms revealed that all investigated variants exhibit a unimodal, right-skewed particle size distribution. Most particles in all tested systems have diameters less than 6 µm. A “long tail” in the distribution was also observed in each of the analyzed groups, indicating a trace presence of larger granules (reaching maximum values of 12–14 µm) (Figure 4, Figure 5 and Figure 6). Furthermore, statistical comparison revealed significant differences in the particle sizes among the formulations, indicating that the choice of wall material and extraction method can influence the final microcapsule dimensions.

### 2.3. HPLC-DAD Analysis

#### 2.3.1. Lutein Concentrations in Extracts

The lutein concentrations in the oil extracts used for microencapsulation, as well as in the final microcapsule powders, were evaluated simultaneously 9 weeks after the microencapsulation process. The highest concentration of lutein was observed in the Soxhlet-extracted sunflower oil sample at 55.25 ± 4.62 µg/g (S_SOX). This was significantly higher (*p* < 0.05) than most other extraction methods, though not statistically distinct from the sunflower oil extraction, which yielded 50.78 ± 0.57 µg/g (S_OIL). Ultrasound-assisted extraction with sunflower oil (S_UAE), as well as extractions with corn oil (C_OIL) and grape seed oil (G_OIL), yielded significantly lower lutein concentrations, ranging from 43.90 ± 2.96 µg/g to 47.90 ± 2.57 µg/g (Table 4).

#### 2.3.2. Lutein Concentrations in Microcapsules

The control sample (CON), produced using the Soxhlet extraction method, sunflower oil, and a gum arabic wall matrix, exhibited the highest final lutein concentration among all tested samples at 32.85 ± 7.18 µg/g. This result was statistically significant compared to the other samples, which utilized different extraction methods and starch-based matrices (Table 5).

Among formulations using starch-based wall materials, those incorporating grape seed oil showed the highest lutein final concentration after 9 weeks. The tapioca starch variant (GTA) yielded a concentration of 26.82 ± 0.77 µg/g, while the waxy maize variant (GHS) yielded 24.97 ± 0.82 µg/g. Formulations prepared by oil extraction with sunflower oil (STA, SHS) and corn oil (CTA, CHS) produced intermediate lutein concentrations, ranging from 19.50 ± 2.69 µg/g to 20.62 ± 0.97 µg/g (Table 6).

## 3. Discussion

### 3.1. Moisture Content

The moisture content of the microcapsules analyzed in the current study ranged from 0.98% to 3.43%. These values demonstrate a high degree of consistency with the existing literature on lutein microencapsulation, particularly the findings of Zhang et al., who reported moisture levels of 0.92–3.50% for lutein stabilized by sodium caseinate and OSA-modified starch [18]. Similarly, the observed results align with research by Zhao et al., in which lutein-loaded powders formulated with whey protein and citric acid potato starch esters maintained moisture levels consistently below 2.50% [19].

In contrast, studies utilizing similar wall material combinations with lipid cores have often reported higher moisture retention. For instance, microcapsules containing medium-chain triglyceride oil and a wall matrix of OSA starch and maltodextrin exhibited moisture contents ranging from 3.68% to 5.23% [14]. Furthermore, significantly higher moisture values, ranging from 4.21% to 9.01%, were observed in formulations containing a mixture of maltodextrin, gum arabic, and modified starch, as in this study. Specifically, a moisture content of 5.68% was recorded for a 50:50 blend of maltodextrin and modified starch, a discrepancy primarily attributed to the high hydration capacity of the starch component in that matrix [17]. In the present study, however, this high hydration capacity was most prominent in the gum arabic control rather than the starch matrices.

This elevation is frequently attributed to high total solid content, which influences the emulsion’s viscosity and can retard water diffusion during the atomization and drying phases [17]. It is possible that the ethanol used in the UAE and Soxhlet extractions co-extracted additional non-lipid solids [20], resulting in a higher total solid content than the pure oil extractions. This likely explains the variations observed between the UAE and pure oil-extracted samples.

Interestingly, 3 out of 4 samples using modified waxy maize starch (EHS, SHS, GHS) achieved the same moisture content, even in the EHS formulation. This suggests that the modified waxy maize starch used in this study yields a repeatable moisture content independently of the core material. The consistency observed in formulations containing waxy maize starch, as well as the differences in moisture retention compared to tapioca starch, likely stem from the distinct physical properties imparted by their respective commercial modification processes. Food industry consensus indicates that maintaining moisture levels below 3–4% is critical for powdered food products, as it can improve microbiological stability, prevent caking, and inhibit the oxidative degradation of sensitive bioactive compounds such as lutein [18].

### 3.2. Solubility

Solubility is an important factor that determines the overall quality of powdered products in the food industry, as it dictates how readily microcapsules dissolve in aqueous food systems.

The statistical analysis revealed that substituting the gum arabic wall material with various starch-based formulations did not significantly affect the powders’ final solubility. This observation strongly aligns with the findings of Fernandes et al. [21], who similarly reported that the type of carbohydrate encapsulant, whether it was pure gum arabic, modified starch, or a mixture, did not significantly alter the solubility of their spray-dried microcapsules. The findings of Pieczykolan and Kurek [22] support it as well. They similarly reported that using different carbohydrate-based coating materials, such as gum arabic, inulin, and pectin, did not result in statistical differences in the water solubility of their microcapsules. Achieving high solubility through spray drying can be explained by the physical mechanisms of the drying process itself. Rapid droplet solidification during the atomization phase produces microcapsules with smooth surfaces, which naturally improve their wettability and dispersion in aqueous systems compared to the irregular, porous structures typically produced by freeze-drying [23].

The solubility values obtained are higher than those in Fernandes et al.’s study, where the highest solubility value was 46.57% [21]. George et al., by encapsulating Moringa oleifera powder in a matrix of maltodextrin and/or gum arabic, obtained higher solubility values ranging from 86.35% to 98.74% [24]. Furthermore, Nawas et al., by encapsulating silver carp oil in a wall material mixture of maltodextrin and gum arabic, achieved a solubility of 89.38% [25]

### 3.3. Surface Oil, Total Oil, and Encapsulation Efficiency

Surface oil content, total oil content, and the resulting encapsulation efficiency (EE) are critical parameters for evaluating the quality of lipid-based microcapsules. Encapsulation efficiency is particularly crucial, as it directly indicates the proportion of the total lipid phase that was successfully entrapped within the polymer matrix. Consequently, it reveals the fraction of unencapsulated surface fat, which remains directly exposed to destructive environmental factors and rapid oxidation [19].

The observed differences in surface oil content may indicate that using starch as a matrix can reduce surface oil content compared to gum arabic. The scientific literature confirms this. In a study by Nawas et al., it was demonstrated that microcapsules produced using OSA-modified starch exhibited lower levels of unencapsulated oil on the surface and significantly higher overall encapsulation efficiency than those based on gum arabic. The authors explain this phenomenon by the fact that chemically modified starches acquire amphiphilic properties, which positively affect their emulsifying properties and their ability to retain oil within the shells during spray drying [25].

The absence of statistically significant differences in total oil content across all tested variants suggests that the starch-based coating modifications exhibit a similar ability to retain total fat mass within the formed structures as the control variant based on gum arabic. Stable total oil values are consistent with the research by Ogrodowska and colleagues, who, during the microencapsulation of pumpkin seed oil using various carbohydrates, recorded total fat content at a very similar, reproducible level, ranging from 32.7% to 36.7% depending on the carbohydrate used [26].

The encapsulation efficiency (EE) obtained in the current study aligns with the findings reported by Ogrodowska et al., who microencapsulated pumpkin seed oil within carbohydrate matrices supplemented with protein concentrate and guar gum. In their study, the EE achieved with microcapsules using rice starch was 35%, and 42% with maize starch. Conversely, significantly higher values were recorded for formulations using maltodextrin (70%) and wheat starch (68%). Our results (34.98–56.59%) fall within this broadly reported range for similar biopolymer matrices [26].

The highest encapsulation efficiency obtained in the current study is remarkably close to the values reported by Álvarez-Henao et al. for spray-dried lutein microparticles. Importantly, their methodology also incorporated a lipid core. In their research, a wall matrix composed of a 1:1 mixture of maltodextrin and modified starch yielded an EE of 56.1%. Similarly, their formulation utilizing a 1:1 mixture of maltodextrin and gum arabic resulted in an EE of 38.8%, which closely mirrors the lower end of the efficiency range observed in our study [14].

Conversely, the encapsulation efficiencies obtained in the current study are lower than those reported by Ding et al., who achieved an EE of 73.2% using a modified starch matrix. However, this discrepancy can be attributed to differences in the core material. Their study used a lutein powder dissolved directly in Tween 80, resulting in a formulation lacking a lipid core.

It should be noted, however, that the encapsulation efficiencies reported in this study were obtained from a single spray-drying batch per formulation, relying entirely on analytical replicates. True independent process replicates could introduce additional variability.

It is worth noting that significantly higher encapsulation efficiencies can be achieved by utilizing combinations of wall materials that incorporate proteins. For example, Zhao et al. microencapsulated lutein using a whey protein-citrate-esterified potato starch matrix, achieving a maximum encapsulation efficiency of 89.36% [19]. Similarly, Zhang et al. combined sodium caseinate with modified starch and achieved efficiencies of 74.7–82.9% [18]. Milk proteins exhibit film-forming properties and help minimize surface cracks on microcapsules, enabling Mora-Gutierrez et al. to achieve a remarkable 97.7% efficiency using caprine casein, yeast beta-glucan, and maltodextrin [27]. Other dairy-derived materials are also highly effective, as shown by Ma et al., who reported an 83.99% efficiency with a mixture of buttermilk powder and gum Arabic, along with greatly improved storage stability [6].

These studies clearly indicate that while pure carbohydrate and gum matrices provide a specific baseline efficiency, the addition of protein-based emulsifiers considerably enhances the structural entrapment and protection of lipid cores.

### 3.4. Bulk Density and Tapped Density

For bulk density, the standout sample is CON, which has a significantly higher bulk density than the others. It also achieved a significantly higher result for tapped density. The remaining starch-based variants were noticeably lighter, with bulk densities ranging from roughly 0.26 to 0.27 g/mL (259.02–272.12 kg/m^3^) and tapped densities between 0.49 and 0.58 g/mL (494.60–587.45 kg/m^3^).

These findings align well with the research by Fernandes et al. [21], whose overall bulk densities ranged from 0.23 to 0.35 g/mL and tapped densities from 0.35 to 0.49 g/mL. Specifically, when they utilized a mixture of modified starch and maltodextrin as the wall material, the resulting microcapsules showed low density values, recording a bulk density of 0.25 g/mL (250 kg/m^3^) and a tapped density of 0.38 g/mL (380 kg/m^3^), which is lower than values achieved by every starch sample in the current study. The highest tapped density in their experiment was observed in the formulation prepared with a mixture of gum arabic and inulin. These results may indicate that the use of gum arabic for the microcapsule shell promotes denser particle packing, unlike starch.

### 3.5. Carr’s Compressibility Index and Hausner Ratio

The conclusion drawn regarding bulk density and tapped density can also be partially applied to flowability indices. The CON formulation still achieved the best processability. However, flowability was not statistically significant across all variants, as seen when compared to ETA, EHS and GTA. Although the CON sample had the best parameters, the microcapsules’ flowability was still virtually none [28]. As mentioned before, the difference in this sample is likely due to the matrix of these microcapsules containing gum arabic, which must have significantly affected the flowability. These results indicate that the analyzed microcapsules exhibit very high cohesiveness, which may negatively affect their use, e.g., in dietary supplements.

Such findings are highly consistent with the research conducted by Napiórkowska et al., who evaluated essential oil microcapsules and reported CI values frequently exceeding 38, with formulations using gum arabic and pea protein as wall materials, and most formulations achieving a Carr Index value of more than 40% [28]. Their study notes that powders obtained by drying processes such as freeze-drying or spray-drying are typically characterized by high cohesiveness and high CI values, which are detrimental to free flow and even distribution during dosing or storage. The results of this study are higher than the indices reported by Areekal et al., whose microencapsulated oil powders exhibited CI values of 30.68–38.33% [29]. While Areekal et al. noted that the inclusion of specific oil blends improved the free-flow behavior of the resulting powders, even their most cohesive pure flaxseed oil formulation demonstrated superior flow characteristics compared to the biopolymer matrices examined in this research.

A similar trend is observed for the Hausner Ratio (HR), with the current experimental values ranging from 1.69 to 2.25, exceeding the range of 1.442 to 1.625 reported for flaxseed oil blends in the Areekal study. However, the achieved ratios closely align with the upper limits reported by Napiórkowska et al., who recorded HR values as high as 2.28 in similar lipid-based systems.

To sum up, these elevated CI and HR values highlight a common phenomenon: spray- or freeze-dried microcapsules often exhibit high interparticulate friction and limited flowability. These properties are typically detrimental to efficient industrial dosing and storage [28].

However, it must be acknowledged that because these flowability indices were derived from a single production run, batch-to-batch variations in droplet formation and particle packing could potentially alter the absolute CI and HR values in independent process replicates.

### 3.6. Surface Morphology of the Microcapsules and Particle Size Distribution

Scanning electron microscopy (SEM) micrographs reveal that the surface morphology and structural integrity of the microcapsules are highly dependent on the specific wall composition. Interestingly, the literature reports contrasting morphological outcomes depending on the specific type of hydrocolloid used. On the one hand, consistent with the observations of San et al., matrices of gum Arabic and maltodextrin can yield a rounder, more spherical geometry compared to the deep surface dents typically seen in specific starch-based matrices, such as octenyl succinic anhydride (OSA) and starch [17]. Conversely, Fernandes et al. demonstrated that combining maltodextrin with a modified starch resulted in a higher proportion of highly spherical particles free of concavities. In contrast, their microcapsules containing gum Arabic and maltodextrin exhibited more surface folds [21].

These morphological variations are fundamentally tied to the drying kinetics and the physical characteristics of the crust. As noted by San et al., surface dents are strongly correlated with prolonged film formation and particle shrinkage during the early stages of spray drying [17]. Thermal expansion of air or steam inside the drying particles can reduce shrinkage, depending on the drying rate and the viscoelastic properties of the matrix material [21].

The particle sizes observed in the current study, with the majority of granules exhibiting diameters below 6 µm and a maximum range of 12–14 µm, are highly consistent with values reported in the recent literature for spray-dried lutein microcapsules. For instance, Álvarez-Henao et al. reported lutein microparticle sizes ranging broadly from 1.64 to 14.20 µm when utilizing various combinations of maltodextrin, gum arabic, and modified starch [14]. Similarly, Ding et al. observed median particle sizes spanning a narrower range of 6.8 to 9.4 µm across formulations employing different carbohydrate matrices, including maltodextrins and modified starch [1]. The slight differences in average size between these studies and the current formulation can be attributed to differences in the viscosity of the feed emulsions, driven by the use of different starches and core-to-wall ratios. Higher viscosities generally lead to the formation of larger droplets during spray atomization and, consequently, larger final dried particles [1]. On one hand, a smaller particle size is advantageous as it enhances solubility by providing a greater contact area within the food matrix; on the other hand, it results in reduced flowability [14].

### 3.7. Lutein Content (HPLC Analysis)

The type of carrier oil significantly influenced final lutein concentration, but interestingly, this effect differed fundamentally between the unencapsulated and encapsulated states. After the 9-week storage period, the unencapsulated sunflower oil extract (S_OIL) exhibited a higher lutein content compared to the corn oil (C_OIL) and grape seed oil (G_OIL) extracts. However, an intriguing inversion was observed within the microencapsulated systems. Microcapsules formulated with a grape seed oil core and a starch matrix (GTA and GHS) achieved the highest final lutein concentration among samples with starch as the wall material.

The lack of consistency between the highest lutein content in the extract and its final concentration in starch-based microcapsules may be due to differences in the oils’ oxidative stability. As demonstrated by Lavecchia and Zuorro, lutein stability is strongly dependent on the type of vegetable oil used, owing to differences in fatty acid profiles and the presence of endogenous antioxidants that protect the sensitive lutein molecule from free radicals generated during lipid peroxidation [30]. The observed discrepancy highlights the complex physicochemical behavior of antioxidants across different multiphasic lipid media. As established in lipid chemistry, extrapolating antioxidant behaviors from bulk oils to emulsified or encapsulated systems is highly inappropriate because the physical location of the oxidation sites changes fundamentally between these states [31].

In the unencapsulated bulk state, lipid oxidation does not primarily occur at the macroscopic air-oil boundary, but rather at the interfaces of nanoscopic “association colloids” (such as reverse micelles) formed by trace water and surface-active minor components within the oil [31,32,33,34]. Grape seed oil is highly susceptible to bulk oxidation due to its high concentration of linoleic acid (68–76%), an n-6 polyunsaturated fatty acid with a low bond dissociation energy, making it highly vulnerable to rapid free radical propagation [35,36]. Conversely, sunflower oil naturally contains exceptionally high levels of lipophilic α-tocopherol, which constitutes roughly 95% of its total endogenous tocopherols [37]. Despite their non-polar nature, highly lipophilic antioxidants like vitamin E can exhibit exceptional antioxidant activity in bulk oils, challenging the classical ‘polar paradox’ due to their specific interactions with association colloids [34].

However, in emulsions, the structural paradigm shifts to a multiphasic environment dominated by a vast oil–water interfacial area, which becomes the primary site of oxidative initiation [31,38]. While current literature predominantly investigates oxidative mechanisms in bulk oils and liquid emulsions, microcapsules are fundamentally dehydrated emulsion systems, and the interfacial partitioning dynamics established for liquid emulsions can be reasonably extrapolated to their solid matrices. Villeneuve et al. emphasize that effective antioxidant activity requires their localization at the interfacial region, where pro-oxidants and lipid hydroperoxides predominantly interact [31].

The highly lipophilic α-tocopherols that dominate sunflower oil are known to partition too deeply into the hydrophobic core of lipid droplets, significantly limiting their availability and efficiency at the droplet interface [31,36]. In this oil, the total tocopherol content ranges from 440 to 1520 mg/kg. There is also significant content of phytosterols and carotenoids. Sunflower oil is not rich in phenols, with an average of about 9.4 mg/kg [37]. By contrast, grape seed oil has a larger hydrophilic fraction, consisting of phenolic compounds such as flavonoids, carotenoids, phenolic acids, tannins, and stilbenes. The range is 59 to 360 mg of gallic acid equivalent/kg of phenols. Among the lipophilic components with antioxidant properties in grape seed oil, the most prominent is vitamin E, present in large amounts, ranging from 1 to 53 mg per 100 g of oil. Grape seed oil is richer in tocotrienols than in tocopherols [39]. In emulsified and encapsulated systems, more polar and amphiphilic phenolic antioxidants migrate naturally toward the oil–water interface [31,38]. Within a microcapsule, it is possible that these phenolics can preferentially migrate toward the polar boundary of the starch wall matrix, establishing a protective interfacial barrier that effectively scavenges aqueous radicals and chelates pro-oxidant metals [31]. Consequently, while the highly lipophilic antioxidant profile of sunflower oil can provide highly effective bulk stabilization, the amphiphilic nature of grape seed oil’s native antioxidants could potentially provide superior interfacial defense. However, it must be noted that this study did not experimentally quantify the specific phenolic profile, antioxidant activity, or the oxidative state of the carrier oils used. Therefore, the migration of amphiphilic phenolics to the oil–water interface remains a literature-based hypothesis to explain the final maximal lutein concentration observed in these starch-based microcapsules, which requires further experimental validation.

Because both the extracts and the microcapsules were analyzed 9 weeks after their initial preparation, their final lutein concentrations reflect different degradation kinetics. The microcapsules were subjected to a high-temperature spray drying process (180 °C inlet temperature) before storage. While this thermal stress can accelerate lutein degradation [40], the heating duration in spray drying is generally brief enough to minimize impact on encapsulated materials [16]. Even though the samples were stored at 4 °C in the dark, which slows the decline in lutein content, complete inhibition of oxidative degradation is not possible [41].

Given this inherent thermal and oxidative stress, it must be acknowledged that direct markers of lipid matrix oxidation (e.g., peroxide value or TBARS) were not explicitly measured in this study, representing a limitation in interpreting the overall oxidative state of the system. However, an analysis of available literature suggests that the spray-drying process does not necessarily lead to the critical degradation of carrier oils. Xu et al. demonstrated that despite short-term thermal stress and a slight initial increase in peroxide value, the fatty acid profile remains stable, and microencapsulation effectively protects the lipid from further rancidity during storage [42]. Furthermore, it has been shown that this technique can significantly increase the oxidative stability index (OSI)—in some cases, nearly doubling it compared to free oil (e.g., from 3.40 h to 6.05 h)—yielding more favorable protective outcomes than freeze-drying [23]. This high efficacy stems from the rapid formation of a dense, spherical shell with a low surface oil content, which acts as a physical barrier that restricts oxygen diffusion and limits the molecular mobility of unsaturated fatty acids. Relying on these documented mechanisms, the present study focused on comparing the relative impact of distinct lipid cores on the preservation of lutein itself. Future studies should, however, incorporate direct carrier oxidation parameters to provide comprehensive insights into the complex oxidative interactions between the lipid matrices and the encapsulated xanthophylls during long-term storage.

Nevertheless, the microcapsules produced in this study exhibited remarkably high final lutein concentrations ranging from 17.62 µg/g to 32.85 µg/g. In contrast, a recent study by Suwanklang et al., which also microencapsulated marigold flower extract within a lipid core, reported lower final lutein contents ranging from 4.69 to 6.18 µg/g. This substantial discrepancy can be attributed to the chemical profile of the carrier oils. While the coconut oil used by Suwanklang provides physical stability due to its saturation [9], the seed oils used in this study possess complex, active profiles of endogenous antioxidants [37,39].

Previous studies have reported that spray-dried emulsions can exhibit compromised lutein stability over time because the microparticle porosity facilitates oxygen penetration, driving continuous degradation during storage [40]. However, SEM analysis shows that the microcapsules made for this study are smooth and continuous. Given the high wall-to-oil ratio used in this study, the resulting barrier is likely thick enough to protect the encapsulated lutein from oxygen exposure. It must be noted, however, that the encapsulation efficiency was relatively low, indicating that only approximately 50% of the lipid phase was effectively encapsulated within the wall material structure. This suggests that the protective capacity of the polymer matrix was limited, resulting in a significant presence of surface oil. Furthermore, a limitation of this study is that the lutein content, measured via HPLC, was determined using three analytical replicates (n = 3) from a single spray-drying batch. Because no independent process replicates were produced, the exact quantitative HPLC results might differ if the entire production process were to be repeated.

Nevertheless, even taking these potential batch-to-batch variations into account, it is important to note that the absolute lutein content in microcapsules can be significantly higher depending on the drying technology used. For instance, Indrawati et al. achieved a massive final lutein ester concentration of 41.19 mg/100 g (411.9 µg/g) in their marigold-derived powder [43]. This exceptional retention was achieved by freeze-drying fresh marigold petals and using freeze-dried encapsulation, thereby eliminating thermal stress.

## 4. Materials and Methods

### 4.1. Materials

The plant material consisted of dried pot marigold (*Calendula officinalis*) petals. Ethanol 99.8% (Chempur, Piekary Śląskie, Poland), sunflower oil (Auchan Polska, Piaseczno, Poland), corn oil (Urzante S.L., Tudela, Spain), and grape seed oil (Kier, Marimax sp. z o.o., Warsaw, Poland) were used as extraction solvents for the initial isolation. Tween 80 (Greenaction sp. z o.o., Kielce, Poland) served as the emulsifier. The wall materials for microencapsulation included maltodextrin (GymBeam, Berlin, Germany), gum arabic (Chempur, Piekary Śląskie, Poland), modified tapioca starch (CAPSUL TA) (Ingredion, Westchester, IL, USA), and modified waxy maize starch (CAPSUL HS) (Ingredion, Westchester, USA).

For the analytical sample preparation and HPLC analysis, the following reagents were used: HPLC-grade methanol (Sigma-Aldrich, Inc., St. Louis, MI, USA), HPLC-grade hexane (Chem-Lab, Piekoszów, Poland), and dichloromethane (DCM) (Chem-Lab, Piekoszów, Poland), Sodium sulfate anhydrous pure p.a. (Chempur, Piekary Śląskie, Poland). Potassium hydroxide (KOH) (Chempur, Piekary Śląskie, Poland) and butylated hydroxytoluene (BHT) (Chem-Lab, Piekoszów, Poland) were used for saponification and stabilization. Other reagents included phosphoric acid (Chempur, Piekary Śląskie, Poland) and sodium dihydrogen phosphate (Chempur, Piekary Śląskie, Poland) for buffer preparation, sodium chloride (NaCl) (Chempur, Piekary Śląskie, Poland), and distilled water. 0.22 µm PTFE syringe filters (Biosens, Warsaw, Poland) were used for filtration.

### 4.2. Sample Preparation

Before extraction, the dried petals were ground in a laboratory grinder (Retsch ZM 200, Retsch Inc., Haan, Germany) to obtain a fraction with a particle size of less than 0.5 mm.

### 4.3. Extraction Procedures

Three different extraction methods were employed. For all extraction variants, the solid-to-solvent ratio was maintained at approximately 15% (*w*/*w*), following the optimized conditions proposed by Manzoor et al. for lutein extraction from marigold petals [13].

- Soxhlet Extraction: The ground petals were extracted with food-grade ethanol using a Soxhlet apparatus. The process lasted 4 h.
- Ultrasound-Assisted Extraction (UAE): This extraction was performed using food-grade ethanol. The process was carried out using an ultrasonic device operating at a frequency of 20 kHz and a power of 70 W. The device was equipped with a 13 mm sonotrode (TS 113, Bandelin, Berlin, Germany) made of a titanium alloy (TiAl6V4), capable of a maximum amplitude of 82 µm. The ultrasonication was conducted for 12.5 min at a set amplitude of 20%. To dissipate the heat generated and prevent the thermal degradation of sensitive bioactive compounds, the extraction vessel was continuously maintained in an ice bath at 4 °C throughout the process.
- Oil Extraction: Sunflower, corn, and grape seed oils were independently used as extraction solvents. For each specific oil, the ultrasound-assisted process parameters were identical to those used in the UAE method.

The unencapsulated liquid extracts were kept at room temperature in transparent bottles under light exposure. These conditions were chosen to simulate real-world commercial practices, mimicking the typical retail storage of edible oils.

Following the Soxhlet and UAE procedures, the ethanolic extracts were subjected to evaporation under reduced pressure to completely remove the solvent. The resulting dry residues were subsequently redissolved in 70 g of sunflower oil. This step created the standardized ‘oil extracts’ that were directly utilized for both the HPLC quantification and the microencapsulation process. In contrast, the extracts obtained via direct oil extraction required no solvent removal and were used as obtained.

### 4.4. Preparation of Emulsions

Lipid matrices were prepared to protect the bioactive compounds. The experimental design included different combinations of wall materials: maltodextrin with gum arabic (control for Soxhlet extract), maltodextrin with tapioca starch, and maltodextrin with waxy maize starch.

The emulsion preparation procedure involved weighing 0.4 g of Tween 80 into a beaker, followed by the preparation of the aqueous phase by mixing polymer solutions. Specifically, 133 g of an aqueous maltodextrin solution (15% *w*/*w*) and 133 g of an aqueous starch or gum arabic solution (15% *w*/*w*) were combined in the same vessel. The mixture was then subjected to magnetic stirring for 10 min. Subsequently, the beaker was placed under a homogenizer, and 20 g of sunflower oil was slowly added while mixing at 5000 rpm. Finally, homogenization was continued at 15,000 rpm for 3 min to obtain the emulsion. Based on the formulation, the total feed solids content of the prepared emulsion prior to spray drying was exactly 21.05% (*w*/*w*).

### 4.5. Microencapsulation

The microencapsulation process was conducted using a Büchi Mini Spray Dryer B-290 (Büchi, Flawil, Switzerland). The spray drying parameters were set as follows: inlet temperature 180 °C, aspirator 100%, actual feed rate of 300 mL/h (corresponding to 30% of the maximum pump capacity of 1 L/h), and a 0.7 mm diameter nozzle with the nozzle cleaner set to 3. Under the applied drying conditions, the outlet temperature was maintained at 85 °C.

The mass of recovered powder was recorded after spray drying for internal process control; however, spray-drying yield was not used as a comparative response variable in this study.

Immediately after the spray-drying process, the obtained microcapsule powders were collected, transferred into screw-capped plastic bottles, and stored in a refrigerator at 4 °C in the dark. This reflects standard industrial protocols for preserving functional ingredients and prevents oxidative or thermal degradation before HPLC analysis (Table 7).

### 4.6. Determination of Physical Properties

#### 4.6.1. Moisture Content

The moisture content (MC) of the powders was determined gravimetrically using the standard oven-drying method (AOAC 925.10, 2005) with slight modifications. Approximately 2.000 g of the sample was accurately weighed into pre-dried and pre-weighed aluminum dishes. The samples were dried in a hot-air oven at 105 ± 2 °C until a constant mass was achieved. Following the drying process, the dishes were transferred to a desiccator containing silica gel, allowed to cool to room temperature for approximately 30 min, and re-weighed. The moisture content was calculated using Equation (1):

(1) $$MC\left(\%\right)\;=\;\frac{m_{i}\;-\;m_{d}}{m_{i}}\;\times \;100$$

where m_i_ represents the initial mass of the sample (g), and m_d_ represents the mass of the sample after drying (g). All measurements were performed in triplicate, and the results were expressed as the mean ± standard deviation.

#### 4.6.2. Solubility

The solubility of the powders was determined using a modified version of the method described by Fernandes et al. [21]. Briefly, 0.100 g of the powder sample was accurately weighed using a precision balance and dispersed in 24.900 g of distilled water within a 50 mL polypropylene tube, yielding a total sample mass of 25 g. To ensure complete dissolution of the soluble fraction, the mixture was gently agitated at room temperature using a tube rotator (MX-RL Pro, DLAB SCIENTIFIC CO., LTD., Beijing, China) at 70 rpm for 30 min.

The dispersion was then centrifuged at 9000 rpm for 10 min at 25 °C. The clear supernatant was carefully collected, and a 10.00 g aliquot was transferred to pre-dried and pre-weighed flasks. The samples were dried in a hot-air oven at 105 °C until a constant mass was achieved (typically 2–3 h), cooled in a desiccator, and weighed. A blank test with distilled water was run in parallel to correct for non-volatile residues.

The mass of the dry residue was used to calculate the solubility, expressed as the percentage of the initially added powder that dissolved in water. Since a 10.00 g aliquot of the total 25.00 g dispersion was analyzed, the solubility (S) was calculated according to the following equation:

(2) $$S\left(\%\right)\;=\;\frac{m_{solut}\;\times \;2.5}{m_{powder}}\;\times \;100$$

where $m_{solut}$ is the mass of the solid residue (g) recovered from the 10 g supernatant aliquot, the factor 2.5 accounts for the total mass of the dispersion (25 g / 10 g), and $m_{powder}$ is the initial mass of the microcapsule powder added (0.100 g). The solubility (S) was expressed as grams of soluble solids per 100 g of water. All measurements were carried out in triplicate, and the results were reported as the mean ± standard deviation.

#### 4.6.3. Surface Oil

The surface oil of the microcapsules was determined by mixing a 1 g portion of the microencapsulated powder with 10 mL of hexane. The dispersion was very gently shaken for 2 min at 25 °C. The mixture was then filtered, and the collected residue was washed with an additional 20 mL of hexane. The resulting filtrate was passed through a funnel containing anhydrous sodium sulfate (98.5%) to remove any residual moisture. Finally, the solvent was evaporated at 60 °C to determine the mass of the surface oil.

#### 4.6.4. Total Oil

The total oil content was determined gravimetrically using a modified method adapted from San et al. [17]. A 1.00 g sample of the powder was dispersed in 5 mL of deionized water and vortexed for 2 min. 25 mL of a solvent mixture containing hexane and propan-2-ol (3:1, *v*/*v*) was added to the aqueous dispersion, and the mixture was extracted using a tube rotator (MX-RL Pro, DLAB SCIENTIFIC CO., LTD., Beijing, China) at a setting of 70 rpm for 5 min.

To separate the phases, the mixture was centrifuged at 3000 rpm for 30 min. The clear upper organic layer was carefully collected. The remaining lower aqueous phase was subjected to a second extraction process using the same volume and composition of the extraction solvent. The organic phases from both extractions were combined and evaporated under a vacuum evaporator (Rotavapor R-300 System Professional, Büchi, Flawil, Switzerland). The mass of the total recovered oil was then determined gravimetrically.

#### 4.6.5. Encapsulation Efficiency

The encapsulation efficiency (EE) of the powders was calculated based on the masses of the total oil and the surface oil, using the following equation:

(3) $$EE\;\left(\%\right)\;=\;\frac{\left(Total\;oil\;-\;Surface\;oil\right)}{Total\;oil}\;\times \;100$$

#### 4.6.6. Bulk Density

The bulk density of the microcapsules was determined according to the procedure described in the Q4B Annex 13, Step 3, with minor modifications. Prior to measurement, samples were equilibrated for at least 30 min at ambient laboratory conditions (23 ± 2 °C) and sieved to remove any agglomerates. Approximately 10 mL of powder was gently poured into a 10 mL graduated cylinder without tapping or compacting. Excess material above the 10 mL mark was carefully leveled using a spatula. The mass of the powder was determined using an analytical balance (±0.1 mg). The bulk density ($\rho_{b}$) was calculated using the following equation:

(4) $$\rho_{b}\;=\;\frac{m}{V}$$

where *m* is the mass of the powder (g) and *V* is the cylinder volume (cm^3^).

#### 4.6.7. Tapped Density

Tapped density was determined manually using a 25 mL graduated cylinder. Approximately 5 g of the powder sample was introduced into the cylinder, and the initial volume ($V_{0}$) was recorded. The cylinder was repeatedly lifted and allowed to fall freely under its own weight onto a solid surface to produce gentle tapping. This process was performed in a series of 50–100 taps until the volume difference between successive readings became negligible (<1%). The final settled volume ($V_{f}$) was recorded, and tapped density was calculated as:

(5) $$\rho_{t}\;=\;\frac{m}{V_{f}}$$

#### 4.6.8. Flowability Indices

To evaluate the flowability, the Carr’s Compressibility Index (CI) and Hausner Ratio (HR) were calculated using the following equations:

(6) $$CI\;=\;\frac{\rho_{t}\;-\;\rho_{b}}{\rho_{t}}\;\times \;100$$

(7) $$HR\;=\;\frac{\rho_{t}}{\rho_{b}}$$

All measurements were performed in triplicate, and results were expressed as mean ± standard deviation.

### 4.7. Scanning Electron Microscopy (SEM)

#### Image Processing and Particle Size Analysis

The morphology of the microcapsules was observed using a Phenom XL (Phenom-World B.V., Eindhoven, The Netherlands) scanning electron microscope, operating at an accelerated voltage of 5 kV, by the method described by Przybył et al. A small amount of the powdered sample was placed on double-sided adhesive tape affixed to the sample support and then flushed with compressed air to remove unattached particles. The sample was subsequently coated with gold using a Cressington 108 auto coater and examined under the SEM [44]. All micrographs were acquired at a uniform magnification of 3000× across all samples to ensure a consistent scale and allow for accurate comparative digital analysis.

Quantitative analysis of the particle size and distribution was performed through digital processing of the acquired SEM micrographs using a custom Python script (Python version 3.12.13). Two representative micrographs were analyzed for each formulation.

The raw .tiff images were first loaded and cropped along the bottom edge (removing 65 pixels) to eliminate the SEM databar and textual annotations, thereby preventing the algorithm from falsely detecting text and scale markers as particles. The cropped images were subsequently subjected to min-max contrast normalization (scaled to a 0–255 intensity range) using the OpenCV library to ensure consistent input for segmentation.

Automated instance segmentation of the individual particles was conducted using Cellpose (version 4.1.1), a deep learning-based segmentation algorithm [45]. The model was executed with GPU acceleration, configured to analyze grayscale inputs (channels = [0, 0]), and optimized with a base object diameter of 30 pixels to effectively separate closely packed granules. As is a known limitation of this algorithm, it inherently struggles to resolve heavily overlapping particles, such as small microcapsules agglomerated on the surface of larger ones. Consequently, these unresolved clusters were not segmented and were excluded from the dataset, ensuring that only distinct, clearly defined particles were analyzed.

Following the generation of instance masks, an algorithmic exclusion criterion was applied to automatically identify and filter out any partially visible microcapsules intersecting the image boundaries, thereby preventing artificial skewing of the size data. The geometric properties of the remaining valid particles were extracted using the NumPy library. The physical dimensions were calibrated based on the SEM scale bar, establishing a spatial resolution of 20 µm per 229 pixels. To determine the pixel length of the scale bar, ImageJ software (version 1.54 g) was used. A straight line was drawn along the scale bar, and the number of pixels was measured using the ‘Set Scale’ function. To address the issue of partial obscuration among the successfully segmented particles, a geometric fitting approach was applied using the OpenCV library. Rather than relying on area-equivalent calculations, the exact diameter of each valid particle was calculated using the minimum enclosing circle method applied to its segmented contour. This mathematical fitting robustly reconstructs the true diameter of spherical particles, effectively compensating for minor lateral overlaps. This pixel-based diameter was subsequently multiplied by the calibration factor to determine the absolute physical diameter of the particles in micrometers (µm).

The numerical data extracted from the segmentation process were utilized to determine the total count of successfully analyzed microcapsules (Valid N) and to calculate the quantitative size descriptors, specifically the mean diameter and standard deviation (mean ± SD). Furthermore, these datasets were used to compute and plot particle size distribution histograms via the Matplotlib library. Additionally, color-coded, semitransparent segmentation masks were overlaid onto the original micrographs to visually verify the accuracy of the automated detection and boundary exclusion.

### 4.8. Chemical Analysis

The content of lutein in the extracts and microcapsules was analyzed using High-Performance Liquid Chromatography with Diode-Array Detection (HPLC-DAD).

#### 4.8.1. Sample Preparation for HPLC Analysis

The extraction of carotenoids from the microcapsules was performed using a saponification method coupled with liquid–liquid extraction, based on the protocol described by Hong et al. [46], with modifications adapted for microencapsulated powders.

0.2 g of microcapsules was weighed into a glass tube. First, 1 mL of deionized water was added and vortexed to dissolve the wall materials. The mixture was sonicated for 3 min (30 °C) to disrupt the matrix.

Next, 3 mL of ethanol containing 0.1% BHT was added. The mixture was vortexed and sonicated for 2 min (30 °C) in the dark.

Saponification was initiated by adding 1.5 mL of 30% KOH in methanol. The samples were incubated for 60 min at ambient temperature (or up to 35 °C) in the dark with gentle shaking. To stop the reaction and enable phase separation, the mixture was acidified by adding approx. 7 mL of 2 M phosphate buffer (pH 2) to reach a weakly acidic pH (approximately 6 pH).

The extraction of carotenoids was performed using a mixture of hexane: dichloromethane (8:2 *v*/*v*) containing 0.1% BHT. A 10 mL volume of the solvent was added, vortexed for 5 min, and centrifuged (9000 rpm, 2 min) to separate the phases. The upper organic layer was collected. This extraction step was repeated three times. The combined organic phases were washed with 10 mL of water (or 15% NaCl solution) to remove alkali residues.

The organic phase was evaporated to dryness under vacuum. The dry residue was redissolved in 2 mL of HPLC-grade methanol. Finally, the solution was filtered through a 0.22 µm PTFE syringe filter into HPLC vials and stored at 4–8 °C in the dark until analysis.

#### 4.8.2. HPLC-DAD Analysis

Lutein was quantified using high-performance liquid chromatography (HPLC) with an external standard method. The analysis was performed on a Shimadzu HPLC system (Shimadzu Corporation, Kyoto, Japan) comprising an LC-20AD pump, SIL-20AC HT autosampler, CBM-20A system controller, SPD-M20A photodiode-array (PDA) detector, and CTO-20A column oven. Chromatographic separation was carried out on a Waters Spherisorb ODS2 column (5 µm particle size, 4.6 × 150 mm) (Waters Corporation, Milford, CT, USA). The column temperature was maintained at 30 °C. The mobile phase consisted of water (solvent A) and methanol (solvent B). The gradient elution program was as follows: 0.01 min, 98% B; 4.50 min, 98% B; 5.00 min, 100% B; 16.00 min, 100% B; 16.01 min, 98% B; 23.00 min, stop. The flow rate was set at 1.0 mL/min. Detection was performed at 450 nm using the PDA detector. Lutein peak identification was confirmed by comparing the retention time with an external reference standard (approximately 3.85 min, with a maximum allowed analytical drift of 0.1 min during a single run). To ensure peak purity and explicitly exclude the interference of co-eluting carotenoids typical for the marigold matrix, the identification was further corroborated by matching the UV-Vis spectral profiles acquired via the PDA detector with the spectrum of the standard. Representative chromatograms and UV-Vis spectra for both the lutein standard and selected sample formulations are provided in the Supplementary Materials. All reagents used for the analysis were of analytical grade and purchased from Sigma-Aldrich (St. Louis, MI, USA). The analysis was performed 9 weeks after encapsulation.

The limit of detection (LOD) and limit of quantification (LOQ) were determined based on the signal-to-noise ratio, using standard analytical criteria: LOD was defined as a signal-to-noise ratio of 3:1, whereas LOQ was defined as a signal-to-noise ratio of 10:1. Based on the calculations performed, the following values were estimated:

- LOD = 0.13 ng/injection
- LOQ = 0.42 ng/injection

For lutein, the calibration curve was linear over the range of 2.45–122.5 ng per injection with four calibration levels, and the equation was f(x) = 6379.25x + 2232.78, with R^2^ = 0.9999964 and RFSD/RFRSD = 8.91%.

#### 4.8.3. Theoretical Lutein Concentration in the Lipid Core

The theoretical concentration of lutein localized within the lipid core of the microcapsules $c_{core}$ (expressed in µg/g of oil) was calculated as an estimate based on the mass balance of the formulation. Because lutein is highly lipophilic and was assumed to be entirely confined to the dispersed oil phase, its concentration in the lipid core was determined using the following equation:

(8) $$c_{core}\;=\;\frac{c_{powder}}{m_{oil}}$$

where $c_{powder}$ is the concentration of lutein quantified in the spray-dried microcapsule powder via HPLC (µg/g of powder), and $m_{oil}$ is the theoretical mass fraction of the oil within the dry microcapsule matrix. The mass fraction ($m_{oil}\;\approx \;0.3317$) was calculated as the ratio of the initial mass of the carrier oil (20 g) to the total dry mass of all solid components in the formulation (60.3 g, comprising wall materials, oil, and emulsifier).

### 4.9. Statistical Analysis

The obtained results were statistically analyzed using Statistica software (Cloud Software Group, Inc., 2023): Data Science Workbench, version 14. It should be noted that each microencapsulated formulation was produced as a single spray-drying batch. Therefore, all experiments were conducted in triplicate (n = 3) representing independent analytical measurements drawn from the same production run. Unless otherwise stated, the reported values represent the mean of these analytical independent experimental replicates. Prior to statistical analysis, the assumptions of normality and homogeneity of variance were verified using the Shapiro–Wilk test and Levene’s test, respectively. The data were subjected to one-way analysis of variance (ANOVA), and the significance of differences between means was determined using Tukey’s HSD post hoc test at *p* ≤ 0.05.

## 5. Conclusions

This study successfully demonstrated the microencapsulation of lutein from marigold (*Calendula officinalis*) using classic Soxhlet, UAE, and solvent-free extraction methods, carrier lipid cores, and polysaccharide wall matrices. The formulated microcapsules exhibited favorable moisture content levels, ranging from 0.98% to 3.43%, which fall well within the industry-recommended range of 3–4% for spray-dried powdered products. Additionally, transitioning to the alternative formulation approaches (featuring modified starch-based materials and different extraction methods) did not significantly alter the solubility of the resulting powders compared to the control, maintaining a consistently high rehydration capacity across all experimental variants.

The microencapsulation process achieved encapsulation efficiencies ranging from 34.98% to 56.59%. Formulations combining waxy maize starch with an oil-extracted sunflower oil core achieved the highest encapsulation efficiency and the lowest unencapsulated surface oil. Morphological analysis via scanning electron microscopy and digital image segmentation confirmed that the microcapsules exhibit a unimodal, right-skewed particle-size distribution, with the vast majority of particles measuring less than 6 µm in diameter. All tested powders exhibited high cohesiveness and poor flowability, as indicated by elevated Carr’s Compressibility Index and Hausner Ratio values, a common challenge for spray-dried microcapsules.

Crucially, this research highlighted the complex, phase-dependent behavior of antioxidants in lipid media. While bulk extraction with sunflower oil yielded the highest lutein concentration, an inversion occurred post-encapsulation, with grape seed oil providing a superior lutein final concentration after 9 weeks within the starch-based microcapsules. Although not explicitly measured in this study, the literature suggests this effect might be due to the amphiphilic phenolic compounds native to grape seed oil migrating to the oil–water interface, potentially forming a protective barrier against oxidative degradation within the dispersed emulsion system. Ultimately, these findings validate the hypothesis of this study: the lipid carrier cannot be treated merely as a passive, non-toxic solvent. Its specific physicochemical and phytochemical profile can significantly differentiate the final lutein content preserved within the spray-dried microcapsules after prolonged storage. Therefore, the rational selection of not only the polymer wall materials but also the core lipid matrix is essential for optimizing powdered dietary supplements. Furthermore, while the solvent-free, non-toxic extraction protocols utilized in this study were efficient at isolating the xanthophyll, the physical limitations of the resulting powders must be addressed. The moderate encapsulation efficiencies and restrictive flowability highlight a clear need for future research to explore more sophisticated encapsulation methods or to incorporate other wall formulations that maximize lutein entrapment and ensure the excellent flow properties required for large-scale supplement manufacturing. Moreover, it must be acknowledged that the final concentration of lutein achieved in these microcapsules remains too low for the production of convenient, single-dose dietary supplements. This limitation stems not only from the encapsulation procedure itself but also fundamentally from the botanical source utilized in this study. While *Calendula officinalis* petals provide effective proof of concept for the extraction and encapsulation mechanisms, future research aiming to achieve commercially viable concentrations should consider alternative botanical sources that offer a significantly higher initial lutein yield.

## Figures and Tables

**Table 1:** Moisture content and solubility of the formulated microcapsules.

Code	Moisture Content [%]	Solubility [%]
CON	3.43 ± 0.06 ^a^	70.5 ± 5.61 ^a^
ETA	2.45 ± 0.05 ^b^	75.59 ± 10.95 ^a^
EHS	1.96 ± 0.04 ^c^	75.84 ± 0.72 ^a^
STA	0.98 ± 0.02 ^e^	80.83 ± 5.35 ^a^
SHS	1.96 ± 0.04 ^c^	75.35 ± 10.68 ^a^
CTA	0.98 ± 0.02 ^e^	81.29 ± 17.11 ^a^
CHS	0.98 ± 0.02 ^e^	85.81 ± 0.24 ^a^
GTA	1.47 ± 0.03 ^d^	75.2 ± 0.24 ^a^
GHS	1.96 ± 0.04 ^c^	80.55 ± 5.52 ^a^

Data are presented as mean ± standard deviation (n = 3 analytical replicates from a single spray-drying batch). Values with different superscript letters in the same column indicate statistically significant differences (*p* ≤ 0.05) according to Tukey’s HSD test. Formulation codes denote the extraction method, oil type, and the additional wall material used in combination with maltodextrin: CON (Soxhlet, sunflower oil, gum arabic); ETA (ultrasound-assisted extraction, sunflower oil, tapioca starch); EHS (ultrasound-assisted extraction, sunflower oil, waxy maize starch); STA (oil extraction, sunflower oil, tapioca starch); SHS (oil extraction, sunflower oil, waxy maize starch); CTA (oil extraction, corn oil, tapioca starch); CHS (oil extraction, corn oil, waxy maize starch); GTA (oil extraction, grape seed oil, tapioca starch); GHS (oil extraction, grape seed oil, waxy maize starch).

**Table 2:** Surface oil, total oil, and encapsulation efficiency of the formulated microcapsules.

Code	Surface Oil [g/100 g]	Total Oil [g/100 g]	Encapsulation Efficiency [%]
CON	20.48 ± 0.50 ^a^	35.67 ± 2.08 ^a^	42.44 ± 4.01 ^ab^
ETA	20.57 ± 1.50 ^a^	31.67 ± 0.58 ^a^	34.98 ± 5.80 ^b^
EHS	18.48 ± 1.50 ^ab^	30.33 ± 4.73 ^a^	37.91 ± 11.89 ^ab^
STA	16.98 ± 1.00 ^abc^	34.33 ± 3.21 ^a^	50.11 ± 7.18 ^ab^
SHS	13.98 ± 3.00 ^c^	32.33 ± 1.53 ^a^	56.59 ± 10.44 ^a^
CTA	17.98 ± 2.00 ^abc^	33.33 ± 1.53 ^a^	45.93 ± 7.43 ^ab^
CHS	15.98 ± 0.04 ^bc^	32.00 ± 1.73 ^a^	49.97 ± 2.57 ^ab^
GTA	18.97 ± 0.04 ^ab^	35.33 ± 4.73 ^a^	45.60 ± 7.86 ^ab^
GHS	16.48 ± 0.50 ^abc^	33.00 ± 3.00 ^a^	49.88 ± 3.07 ^ab^

Data are presented as mean ± standard deviation (n = 3 analytical replicates from a single spray-drying batch). Values with different superscript letters in the same column indicate statistically significant differences (*p* ≤ 0.05) according to Tukey’s HSD test. Formulation codes denote the extraction method, oil type, and the additional wall material used in combination with maltodextrin: CON (Soxhlet, sunflower oil, gum arabic); ETA (ultrasound-assisted extraction, sunflower oil, tapioca starch); EHS (ultrasound-assisted extraction, sunflower oil, waxy maize starch); STA (oil extraction, sunflower oil, tapioca starch); SHS (oil extraction, sunflower oil, waxy maize starch); CTA (oil extraction, corn oil, tapioca starch); CHS (oil extraction, corn oil, waxy maize starch); GTA (oil extraction, grape seed oil, tapioca starch); GHS (oil extraction, grape seed oil, waxy maize starch).

**Table 3:** Bulk density, tapped density, Carr’s Compressibility Index, and Hausner Ratio for microcapsules.

Code	Bulk Density [kg/m^3^]	Tapped Density [kg/m^3^]	Carr’s Index [%]	Hausner Ratio
CON	394.66 ± 5.90 ^b^	666.00 ± 1.15 ^a^	40.70% ± 0.88% ^c^	1.69 ± 0.03 ^c^
ETA	267.65 ± 3.75 ^a^	506.73 ± 50.59 ^c^	46.87% ± 4.66% ^bc^	1.90 ± 0.17 ^bc^
EHS	260.47 ± 30.96 ^a^	494.60 ± 4.98 ^c^	47.35% ± 5.73% ^bc^	1.92 ± 0.21 ^bc^
STA	261.74 ± 4.59 ^a^	524.60 ± 30.28 ^bc^	49.92% ± 2.02% ^ab^	2.00 ± 0.08 ^ab^
SHS	264.19 ± 4.08 ^a^	553.12 ± 27.57 ^bc^	52.08% ± 1.65% ^ab^	2.09 ± 0.07 ^ab^
CTA	259.02 ± 1.53 ^a^	582.59 ± 33.94 ^b^	55.33% ± 2.36% ^a^	2.25 ± 0.12 ^a^
CHS	271.58 ± 6.49 ^a^	540.21 ± 14.67 ^bc^	49.66% ± 0.20% ^ab^	1.99 ± 0.01 ^abc^
GTA	271.14 ± 1.88 ^a^	525.98 ± 13.91 ^bc^	48.36% ± 1.03% ^abc^	1.94 ± 0.04 ^bc^
GHS	272.12 ± 2.75 ^a^	587.45 ± 1.36 ^b^	53.61% ± 0.47% ^ab^	2.16 ± 0.02 ^ab^

Data are presented as mean ± standard deviation (n = 3 analytical replicates from a single spray-drying batch). Values with different superscript letters in the same column indicate statistically significant differences (*p* ≤ 0.05) according to Tukey’s HSD test. Formulation codes denote the extraction method, oil type, and the additional wall material used in combination with maltodextrin: CON (Soxhlet, sunflower oil, gum arabic); ETA (ultrasound-assisted extraction, sunflower oil, tapioca starch); EHS (ultrasound-assisted extraction, sunflower oil, waxy maize starch); STA (oil extraction, sunflower oil, tapioca starch); SHS (oil extraction, sunflower oil, waxy maize starch); CTA (oil extraction, corn oil, tapioca starch); CHS (oil extraction, corn oil, waxy maize starch); GTA (oil extraction, grape seed oil, tapioca starch); GHS (oil extraction, grape seed oil, waxy maize starch).

**Table 4:** Diameter of microcapsules.

Code	Valid N	Diameter of Microcapsule [µm]	D_10_ [µm]	D_50_ [µm]	D_90_ [µm]
CON	1090	4.07 ± 1.90 ^cd^	2.06	3.66	6.58
ETA	1040	4.39 ± 2.25 ^b^	2.22	3.86	7.39
EHS	1135	4.11 ± 2.01 ^bcd^	2.16	3.57	6.48
STA	676	4.33 ± 2.52 ^bc^	2.01	3.52	7.54
SHS	796	4.34 ± 2.24 ^bc^	2.11	3.75	7.58
CTA	906	4.38 ± 2.23 ^b^	2.15	3.81	7.42
CHS	889	4.80 ± 2.28 ^a^	2.55	4.37	7.56
GTA	1052	4.22 ± 2.00 ^bc^	2.23	3.71	6.63
GHS	1165	3.85 ± 1.94 ^d^	1.99	3.36	6.36

Data are presented as mean ± standard deviation (n = 3 analytical replicates from a single spray-drying batch). Values with different superscript letters in the same column indicate statistically significant differences (*p* ≤ 0.05) according to Tukey’s HSD test. Formulation codes denote the extraction method, oil type, and the additional wall material used in combination with maltodextrin: CON (Soxhlet, sunflower oil, gum arabic); ETA (ultrasound-assisted extraction, sunflower oil, tapioca starch); EHS (ultrasound-assisted extraction, sunflower oil, waxy maize starch); STA (oil extraction, sunflower oil, tapioca starch); SHS (oil extraction, sunflower oil, waxy maize starch); CTA (oil extraction, corn oil, tapioca starch); CHS (oil extraction, corn oil, waxy maize starch); GTA (oil extraction, grape seed oil, tapioca starch); GHS (oil extraction, grape seed oil, waxy maize starch).

**Table 5:** Concentration of lutein in oil extracts.

Code	Concentration of Lutein in the Sample [µg/g]
S_SOX	55.25 ± 4.62 ^a^
S_UAE	43.9 ± 2.96 ^b^
S_OIL	50.78 ± 0.57 ^ab^
C_OIL	47.9 ± 2.57 ^b^
G_OIL	46.47 ± 3.87 ^b^

Data are presented as mean ± standard deviation (n = 3 analytical replicates from a single extraction batch). Values with different superscript letters in the same column indicate statistically significant differences (*p* ≤ 0.05) according to Tukey’s HSD test. Extraction codes: S_SOX (Soxhlet extraction using ethanol); S_UAE (ultrasound-assisted extraction using ethanol); S_OIL (oil extraction using sunflower oil); C_OIL (oil extraction using corn oil); G_OIL (oil extraction using grape seed oil).

**Table 6:** Concentration of lutein in microcapsules.

Code	Concentration of Lutein in the Sample [µg/g]	Lutein Concentration in the Lipid Core [µg/g]
CON	32.85 ± 7.18 ^a^	99.04 ± 21.64 ^a^
ETA	18.8 ± 3.27 ^cd^	56.68 ± 9.86 ^cd^
EHS	17.62 ± 0.54 ^d^	53.11 ± 1.63 ^d^
STA	20.07 ± 1.02 ^bcd^	60.5 ± 3.06 ^bcd^
SHS	20.62 ± 0.97 ^bcd^	62.15 ± 2.91 ^bcd^
CTA	19.5 ± 2.69 ^cd^	58.79 ± 8.1 ^cd^
CHS	20.12 ± 1.45 ^bcd^	60.65 ± 4.37 ^bcd^
GTA	26.82 ± 0.77 ^ab^	80.85 ± 2.31 ^ab^
GHS	24.97 ± 0.82 ^bc^	75.27 ± 2.46 ^bc^

Data are presented as mean ± standard deviation (n = 3 analytical replicates from a single spray-drying batch). Values with different superscript letters in the same column indicate statistically significant differences (*p* ≤ 0.05) according to Tukey’s HSD test. Formulation codes denote the extraction method, oil type, and the additional wall material used in combination with maltodextrin: CON (Soxhlet, sunflower oil, gum arabic); ETA (ultrasound-assisted extraction, sunflower oil, tapioca starch); EHS (ultrasound-assisted extraction, sunflower oil, waxy maize starch); STA (oil extraction, sunflower oil, tapioca starch); SHS (oil extraction, sunflower oil, waxy maize starch); CTA (oil extraction, corn oil, tapioca starch); CHS (oil extraction, corn oil, waxy maize starch); GTA (oil extraction, grape seed oil, tapioca starch); GHS (oil extraction, grape seed oil, waxy maize starch).

**Table 7:** Formulation codes and ingredient quantities used per single batch.

Code	Type of Extraction	Type of Oil Used	Oil Extract [g]	Tween 80 [g]	15% Maltodextrin Solution [g]	15% Gum Arabic Solution [g]	15% Tapioca Starch Solution [g]	15% Waxy Maize Starch Solution [g]
CON	Soxhlet	Sunflower	20	0.4	133	133	-	-
ETA	UAE ^1^	Sunflower	20	0.4	133	-	133	-
EHS	UAE ^1^	Sunflower	20	0.4	133	-	-	133
STA	Oil extraction	Sunflower	20	0.4	133	-	133	-
SHS	Oil extraction	Sunflower	20	0.4	133	-	-	133
CTA	Oil extraction	Corn	20	0.4	133	-	133	-
CHS	Oil extraction	Corn	20	0.4	133	-	-	133
GTA	Oil extraction	Grape seed oil	20	0.4	133	-	133	**-**
GHS	Oil extraction	Grape seed oil	20	0.4	133	-	-	133

^1^ Ultrasound-Assisted Extraction.

## Data Availability

The original contributions presented in the study are included in the article, further inquiries can be directed to the corresponding author.

## Supplementary Materials

The following supporting information can be downloaded at: https://www.mdpi.com/article/10.3390/molecules31101649/s1.
