# Supplementary material for: Solvent-Free Oil-Based Extraction and Microencapsulation of Lutein from Marigold (Calendula officinalis)
Source: Molecules. 2026 May 13;31(10):1649. doi: 10.3390/molecules31101649 (PMC13209855; doi:10.3390/molecules31101649)

UV-Vis spectrum of the lutein reference standard

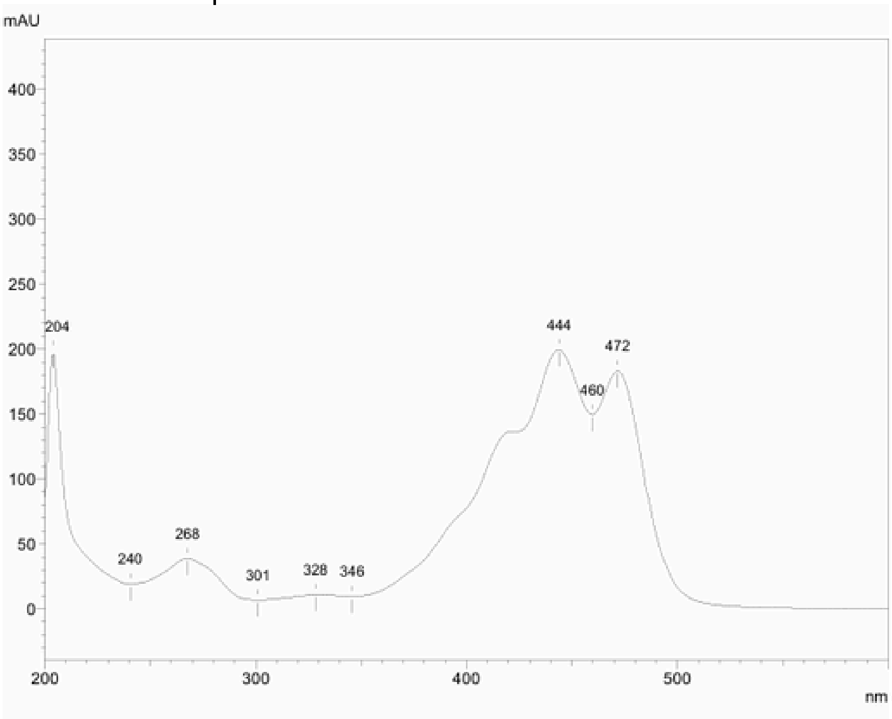

UV-Vis spectrum of the corresponding sample peak, verifying the analyte's identity against the standard

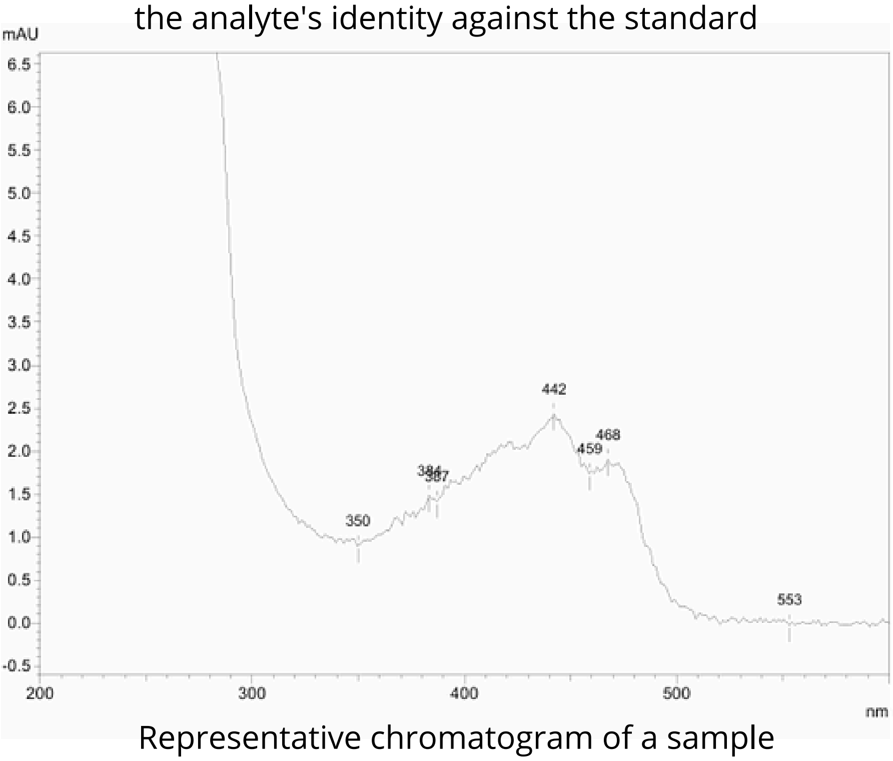

Representative chromatogram of a sample formulation showing the lutein peak at RT 3.841 min

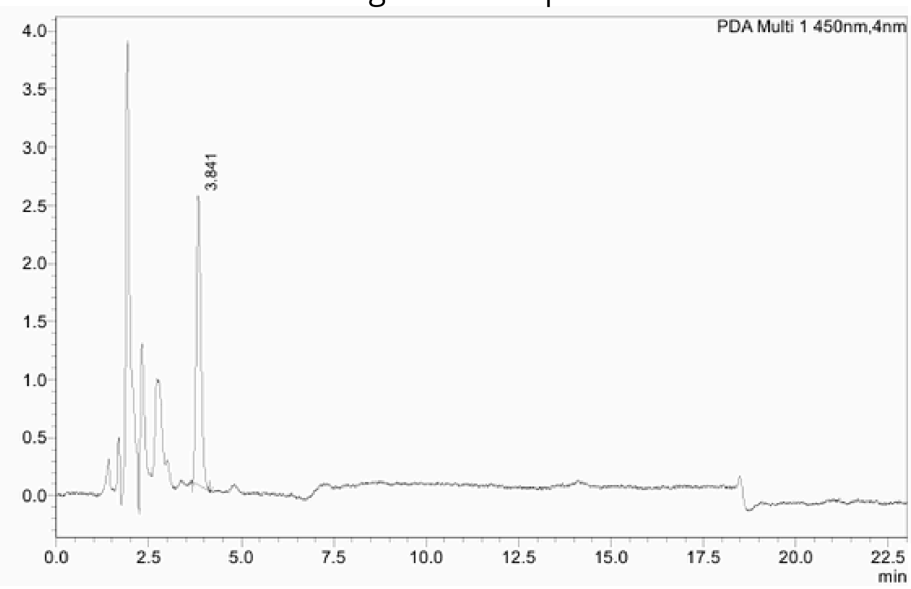

Supplement: Supplementary file 1 [file molecules-31-01649-s001.zip › molecules-4279098-supplementary.pdf]
